# Reducing inequities in maternal and child health in rural Guatemala through the CBIO+﻿ Approach of Curamericas: 10. Summary, cost effectiveness, and policy implications

**DOI:** 10.1186/s12939-022-01762-w

**Published:** 2023-02-28

**Authors:** Henry B. Perry, Ira Stollak, Mario Valdez

**Affiliations:** 1 Health Systems Program, Department of International Health, Johns Hopkins Bloomberg School of Public Health, Baltimore, Maryland USA; 2 Curamericas Global, Raleigh, North Carolina USA; 3 Curamericas/Guatemala, Quetzaltenango, Guatemala

**Keywords:** Maternal health, Child health, Community health, Primary health care, Community-based primary health care, Implementation research, Census-Based, Impact-Oriented Approach, Care Groups, Community Birthing Centers, Guatemala, Equity, Curamericas Global, Curamericas/Guatemala

## Abstract

**Background:**

This is the final of 10 papers that describe the implementation of the Expanded Census-Based, Impact-Oriented Approach (CBIO+) by Curamericas/Guatemala in the Cuchumatanes mountains of the Department of Huehuetenango and its effectiveness in improving the health and well-being of women and children in a population of 98,000 in three municipalities. The CBIO+ Approach consists of three components: the CBIO (Census-Based, Impact-Oriented) Approach, the Care Group Approach, and the Community Birthing Center Approach.

**Methods:**

Each of the preceding papers was summarized. An assessment was made regarding the degree to which the initial implementation research hypotheses were confirmed. The total field cost per capita for operation of the Project was calculated. An assessment of the cost-effectiveness of the Project was made based on the estimated impact of the Project, the number of lives saved, and the number of disability-adjusted life years averted.

**Results:**

The Project attained a number of notable achievements in terms of expanding the coverage of key maternal and child health interventions, improving the nutritional status of children, reducing the mortality of children and mothers, providing quality care for mothers at the Community Birthing Centers (*Casas Maternas Rurales*) that integrate traditional midwives (*comadronas*) into the care of women during childbirth at the birthing centers, as well as empowering women and building social capital in the communities. CBIO+ is an effective and affordable approach that is particularly notable for its capacity to engage communities in the process of improving the health of mothers and children. Overall, there is strong and consistent evidence in support of the research hypotheses. The findings did produce evidence of declines in under-5 and maternal mortality, but they were not as robust as had been hoped.

**Conclusion:**

CBIO+ is an approach that has been effective in engaging communities in the process of improving the health of their mothers and children and in reducing health inequities in this marginalized, difficult-to-reach population of Indigenous Maya people. The CBIO+ Approach is cost-effective and merits further development and broader application in Guatemala and beyond.

## Background

This is the final paper in our series of 10 papers that describe the implementation of the Expanded Census-Based, Impact-Oriented Approach (CBIO+) by Curamericas/Guatemala in the Cuchumatanes mountains of the Department of Huehuetenango and its effectiveness in improving the health and well-being of women and children in a population of 98,000 in three municipalities: Santa Eulalia, San Miguel Acatán, and San Sebastián Coatán. The Maternal and Child Health Project, 2011–2015 (hereafter referred to as the Project) was implemented from October 2011 until May 2015 in Area A, comprising approximately one-half of the Project population, and from October 2013 until May 2015 in Area B. Our purpose here is to (1) briefly summarize each of the preceding papers, (2) review the initial implementation research hypotheses and the extent to which our research confirmed these hypotheses, (3) assess the costs and cost-effectiveness of the CBIO+ Approach, and, finally, (4) consider the broader implications of our work.

The CBIO+ Approach is an expansion of CBIO and is composed of three components: (2) the Census-Based, Impact-Oriented (CBIO) Approach, (2) the Care Group Approach, and (3) the Community Birthing Center Approach. The CBIO Approach consists of conducting a census with the community, registering all households, identifying local epidemiological priorities –the most frequent and serious readily preventable or treatable conditions in the population – and the health priorities according to the local people, developing a plan to address these priorities, and assessing over time whether the health of the population has improved [1]. All of this is accomplished through (1) partnerships with the community, (2) collection of local data, and (3) routine systematic home visitation to collect data, including vital events, and to deliver services. Further descriptions of the approach and its effectiveness are available [2–6].

The Care Group Approach is an extension of CBIO that involves the selection of one female Care Group Volunteer for every 10–15 households with a pregnant woman or mother of an under-2 child. Then, 5–12 Care Group Volunteers meet with a Care Group Promoter every 2–4 weeks to learn 1–2 educational messages to share with the mothers in the assigned households area for each Care Group Volunteer, either by visiting each home separately or meeting as a group. The Care Group Approach is a pedagogic model that utilizes cascaded learner-empowered participation to actively engage participants in the learning process. Its lessons are designed for non-literate audiences and teachers. At the subsequent meeting, the Promoter teaches them a new message and the Care Group Volunteers report pregnancies, births and deaths to the Promoter [7]. A broad body of experience and evidence from implementation by many organizations throughout the world now supports the effectiveness of the Care Group Approach in achieving household-level behavior change resulting in high coverage of key interventions for maternal and child health as well as in improvements in child nutrition and under-5 mortality. The Care Group model was developed in Mozambique by the international non-governmental organization (NGO) World Relief [7]. Curamericas piloted the combined CBIO and Care Group methodologies from 2002 to 2007 in Guatemala and later in Liberia between 2008 and 2013. These pilots demonstrated that Care Group Volunteers could report vital events and achieve high population coverage of evidence-based interventions for improving maternal and child health [8, 9].

The Community Birthing Center Approach as developed by Curamericas/Guatemala is a participatory approach that involves working with communities to construct, staff and operate a readily available local site where mothers can safely give birth in a way that respects traditional customs and enables the traditional midwife (called a *comadrona* in the Project Area) to perform her traditional role. These centers are staffed 24/7 by auxiliary nurses with special additional training and supervised by an experienced obstetrical graduate nurse who is based at one of the birthing centers and is available by phone to support the other birthing centers. Connected to each birthing center is an emergency transport system to provide prompt referral to a hospital should the need arise. Also associated with the birthing center is an insurance system that pregnant women and their families can pay into during the pregnancy to offset to cost of transport if a referral is needed [10]. We are not aware of any other published reports other than our own report in this series that describe the management of obstetrical complications at similar community birthing centers elsewhere.

## Methods

We first reviewed the initial nine papers of this series [11–19] and the original implementation research hypotheses [12]. We then summarized the main findings and the degree to which the hypotheses were confirmed. We summarized the costs of Project implementation, our estimate of the number of deaths of mothers and children averted by the Project, and the number of years of life saved. From these statistics we then calculated the number of disability-adjusted-life-years (DALYs) averted and the cost per DALY averted. Finally, we drew on these findings to describe the broader implications of our work.

## Results

### Key findings from papers 1–9

Table 1 contains the main findings of each of the preceding papers. Taken together, the Project achieved a number of notable achievements in terms of expanding the coverage of key maternal and child health interventions, improving the nutritional status of children, reducing the mortality of children and mothers, providing quality care for mothers at the Community Birthing Centers﻿ as well as empowering women and building social capital in the communities. Care Groups provided the opportunity for mothers to work together to change household behaviors, nutritional practices and health care utilization that led to readily observed health benefits, thereby serving as a powerful platform for strengthening women’s empowerment and social capital in the community. CBIO+ was considered to be an effective and feasible approach that was particularly notable for its capacity to engage communities in the process of improving the health of mothers and children.

**Table 1:** Key findings of Papers 1–9

Paper number and reference	Title	Key findings
1 [11]	Introduction and project description	The components of CBIO+ (CBIO, Care Groups, and Community Birthing Centers) have a long history of effective implementation but have not been widely adopted. This is the first example we are aware of the three components having been implemented together and evaluated. The Project Area was a challenging one in terms of the mountainous terrain, the difficulty of geographic access, the traditional culture and language barriers, and the history of conflict during the Guatemala civil war (1960–1996)
2 [12]	Study site, design and methods	The implementation research consisted of multiple household surveys at baseline and endline in both Areas A and B, numerous in-depth individual and group interviews, focus group discussions, and the registration of vital events. Since interventions in Area B began midway through the implementation of the Project, it served as a quasi-control area
3[13]	Expansion of population coverage of key interventions	Statistically significant improvements were observed in the coverage in 21 of 24 evidence-based interventions in Area A and 19 of 24 evidence-based interventions in Area B. There was a three-fold (200%) increase for 7 of 24 indicators in Area A and for 5 of 24 indicators in Area B. There was no improvement for indicators of interventions that required support from the government’s Extension of Coverage (PEC) Program (immunizations, vitamin A, and family planning) as a result of the government's shutdown of its PEC Program during the Project implementation
4 [14]	Nutritional assessment	Levels of stunting in under-2 children in Area A declined from 74.5% to 39.5%, with endline levels considerably lower than for comparison areas outside of the Project Area. The endline level of stunting in Area B was lower than for comparison areas outside of the Project Area but higher than in Area A. Improvements in multiple output and outcome indicators associated with nutritional status were also observed in Areas A and B. These included infant and child feeding practices, routine growth monitoring and counseling, and household practices for the prevention and treatment of diarrhea. The results were stronger in Area A
5 [15]	Mortality assessment	The maternal mortality ratio declined from 632 in Years 1 and 2 to 257 per 100,000 live births in Years 3 and 4 in Area A (*p*=.006). There was no decline in the under-5 mortality rate (U5MR) in Area A (45 per 1,000 live births in Years 1 and 2 and 45 per 1,000 live births in Years 3 and 4). The 12-<60-month mortality rate declined from 9 deaths per 1,000 live births in the first three years of the Project to 2 in the final year. No declines in mortality were observed in Area B, where the Project operated for only 15 months in Years 3 and 4. Incomplete registration of deaths during the first two years of Project operations appears to have muted the mortality impact of the Project as measured by vital events registration. An indirect estimate of mortality declines using the Lives Saved Tool (based on changes in population coverage of evidence-based interventions) suggests a net decline, independent of ongoing secular changes, of 12% for maternal mortality and 22% for under-5 mortality
6 [16]	Management of pregnancy complications at Community Birthing Centers	15% of 1,378 women coming to a birthing center between 2009 and 2016 experienced a complication; 42% were managed successfully at the birthing center and 58% were referred to a higher-level facility. Only one maternal death occurred. Referrals were rejected initially by the patient or the family in approximately 15% of cases but eventually almost all accepted the recommendation. Birthing Center staff attributed their successful management of complications to intensive training, teamwork, and logistical support
7 [17]	The empowering effect of Care Groups	Participation in the Care Group process was an empowering process for women. Mothers reported increased respect accorded to them by the community, an increased willingness and ability to make health-related decisions, as well as the development of stronger bonds among Care Group members and with other community members and community leaders
8 [18]	Empowerment of women	Household surveys revealed statistically significant increases in women’s active participation in community meetings and in health-related decision-making. These findings corroborated qualitative findings from focus group discussions that the Project has accelerated progress in increasing women’s empowerment, though women still face major barriers in accessing needed health care services for themselves and their children
9 [19]	Key stakeholder perspectives on CBIO+	Project staff members and government health workers were enthusiastic supporters of CBIO+ , especially its approach to involving the community in program planning. There was a strong desire among government health workers for the Project to continue

### Review of implementation research findings in relation to the original research hypotheses

In Table 2 we list the original research hypotheses and state the main findings of our research as they relate to these hypotheses. Overall, there was strong and consistent evidence in support of the research hypotheses. The findings did produce evidence of declines in under-5 and maternal mortality, but they were not as robust as had been hoped.

**Table 2:** The research hypotheses and the research findings related to them

Hypothesis	Outcomes	Location of details
The CBIO+ Approach improves the population coverage of interventions that are designed to address the epidemiological priorities for mothers and children relative to (a) baseline measures of these indicators, (b) measures in a comparison area (Area B), (c) measures in selected nearby municipalities where the Project was not in operation, and (d) the overall rural population of the Department of Huehuetenango	There was a statistically significant increase in the population coverage of 19 of the 24 indicators under the Project’s control in Area A and 19 of the 24 in Area B. Coverage improvements were greater in Area A than in Area B for 8 of these 24 indicators, but improvements in Area B were surprisingly strong given the shorter period of implementation. We were not able to locate appropriate comparable data for adjacent municipalities nor for the rural population of the Department of Huehuetenango. However, we were able to compare our findings for 17 indicators with those for the entire Department of Huehuetenango. For 7 of the 17 indicators, the levels of coverage were higher in the Project Area in spite of the fact that the Project Area was one of the most isolated and impoverished sections of the Department and the data for the Department as a whole included urban areas as well	Paper 3 [13]
The CBIO+ Approach improves the nutritional status of children relative to (a) baseline measures of these indicators, (b) measures in a comparison area (Area B), (c) measures in selected nearby municipalities where the Project was not in operation, and (d) the overall rural population of the Department of Huehuetenango	Significant improvements in stunting and underweight were observed in Area A. The improvements were greater in Area A than in Area B. Comparisons with the nutritional data for the rural population of the Department of Huehuetenango and for the Northwest Region of Guatemala revealed lower levels of stunting in the Project Area at endline	Paper 4 [14]
The CBIO+ Approach reduces under-5 mortality and maternal mortality relative to (a) baseline measures of these indicators, (b) measures in a comparison area (Area B), (c) measures in selected nearby municipalities where the Project was not in operation, (d) the overall rural population of the Department of Huehuetenango	The mortality impact assessment based on vital events data demonstrated a statistically significant decline in maternal mortality and in 12-<60-month mortality but not in neonatal or infant mortality. It appears that the measured baseline levels were artificially low because of incomplete registration of deaths. However, when we applied the Lives Saved Tool, which estimates mortality impact based on changes in coverage of key evidence-based indicators, there was a 22% decline in under-5 mortality and 12% decline in maternal mortality relative to projected ongoing declines in the absence of the Project	Paper 5 [15]
The Birthing Center Approach provides mothers with a safe alternative to home delivery that is also culturally appropriate and attractive to mothers, their families, and to their *comadronas* (traditional midwives)	Review of the maternal mortality data and clinical records of patients giving birth at birthing centers all confirm the greater safety of a delivery at a birthing center compared to a home birth as well as its attraction for mothers and *comadronas*	Papers 1 and 7 [11, 17]
The CBIO+ Approach empowers women engaged as volunteers and as beneficiaries, and it improves self-esteem and decision-making autonomy. The CBIO+ Approach also builds social capital	Interviews with Care Group participants provided numerous examples in which their participation led to increased respect from others in the community, increased willingness and ability to make decisions, and even stronger bonds with other Care Group members, community members, and community leaders. Household survey data revealed increases in women’s active participation in community meetings and in health-related decision-making in the home	Papers 7 and 8 [17, 18]
Stakeholders, including Project beneficiaries, community leaders, Project staff, and MSPAS staff, consider the CBIO+ Approach to be effective and appropriate for improving the health and well-being of children and their mothers	Stakeholders were highly supportive of the CBIO+ Approach, the manner in which it was implemented, and the added value that it brought to the MSPAS health system. Among the many benefits mentioned was the value of engaging the community in the process of improving the health of mothers and children	Paper 9 [19]

*MSPAS Ministerio de Salud Pública y Asistencia Social* (Ministry of Health and Social Assistance)

### Cost and cost-effectiveness analysis

#### How much did the project cost?

As shown in Table 3, the total field costs of Project activities over the four years of implementation were $1.515 million. The average cost per beneficiary for one year of Project operations was $14.05, and the average cost per capita for one year of Project operations was $5.80. Birthing center expenses were 14.0% of the Project field expenses.

**T﻿able 3:** Population served, program expenses, and costs per capita and per beneficiary

**Project Year**	**Number of beneficiaries and total population**			**Number of functioning birthing centers**	**Project field expenses**	**Annualized field expenses**	
	**Project Area A (Phase I communities)**	**Project Area B (Phase II communities)**	**Total Project Area (A & B combined)**			**Per beneficiary**	**Per capita (total population)**
1 (Oct. 2011-Sept. 2012)	WRA: 10,532 U-5: 6,716 Total beneficiaries: 17,248 Total population: 42,755	0	WRA: 10,532 U-5: 6,716 Total beneficiaries: 17,248 Total population: 42,755	1	$198,606	$11.51	$4.65
2 (Oct. 2012-Sept. 2013)	WRA: 10,532 U-5: 6,716 Total beneficiaries: 17,248 Total population: 42,755	0	WRA: 10,532 U-5: 6,716 Total beneficiaries: 17,248 Total population: 42,755	2	$277,808	$16.11	$6.50
3 (Oct. 2013-Sept. 2014)	WRA: 11,159 U-5: 6,636 Total beneficiaries: 17,795 Total population: 43,563	WRA: 12,285 U-5: 6,496 Total beneficiaries: 18,781 Total population: 44,335	WRA: 23,444 U-5: 13,132 Total beneficiaries: 36,576 Total population: 87,898	3	$466,675	$12.76	$5.31
4 (Oct. 2014-Sept. 2015)	WRA: 11,283 U-5: 6,971 Total beneficiaries: 18,254 Total population: 44,479	WRA:11,848 U-5: 6,667 Total beneficiaries: 18,515 Total population: 43,159	WRA: 23,131 U-5: 13,638 Total beneficiaries: 36,769 Total population: 87,638	3	$571,986	$15.56	$6.53
Total	WRA person-years: 43,506 U-5 person-years: 27,039 Total beneficiary person-years: 70,545 Total population person-years: 173,552	WRA person-years: 24,133 U-5 person-years: 13,163 Total beneficiary person-years: 37,296 Total population person-years: 87,494	WRA person-years: 67,729 U-5 person-years: 40,202 Total beneficiary person-years: 107,931 Total population person-years: 261,046	3	$1,515,075	$14.05	$5.80

*WRA* Women of reproductive age, *U-5* Children younger than 5 years of age; Annual cost per capita is the average cost per person﻿ for the entire population of all age groups; Total number of person-years are calculated by adding up the populations for each year of program participation

#### How many lives did the project save and at what cost?

As shown in Table 4, we estimate using the Lives Saved Tool (LiST) that the Project would have saved under-5 lives per year and two maternal lives per year when implemented in the entire Project Area at an annual cost of $5.80 per capita per year or $568,400 per year for 98,000 people. As shown in Table 4, this leads to an estimated cost of $17,224 per year for each life saved.

**Table 4:** Lives Saved Tool (LiST) estimates of the number of lives saved each year and cost per disability-adjusted life year (DALY) averted by the Curamericas/Guatemala Maternal and Child Health Project, 2011–2015

**Number of child lives saved each year**						
Decline of under-5 mortality:	Estimated U5MR at baseline:	Estimated U5MR in Year 4:	Number of births per year:	Number of deaths per year if U5MR were 54:	Number of deaths per year if U5MR were 42:	Difference (number of lives saved per year):
22%	54 deaths per 1,000 live births	42 deaths per 1,000 live births	2,575	139	108	31
**Number of maternal lives saved each year**						
Decline of maternal mortality:	Estimated MMR at baseline:	Estimated MMR in Year 4:	Number of births per year:	Number of deaths if MMR were 632:	Number of deaths if MMR were 556:	
12%	632 deaths per 100,000 live births	556 deaths per 100,000 live births	2,575	16	14	2
**Total number of lives saved each year**						33
**Total Project cost for one year of operation**^**a**^						$568,400
**Cost per life saved**						$17,224
**Number of years of life saved**^**b**^						
	66 years for each under-5 death averted	31*66 = 2,046	50 years for each maternal death averted	2*50 = 100	Total = 2,208	2,146
**Cost per year of life saved**						$265
**Number of DALYs averted each year**^**c**^						2,146
**Cost per DALY averted**						$265

*U5MR* Under-5 mortality rate, *MMR* Maternal mortality ratio, *DALY* Disability-adjusted life-year

^a^Based on a per-capita cost of $5.80 for a population of 98,000

^b^The life expectancy at birth for Guatemala in 2015 was 71 years [23]. For these calculations we assume conservatively that each child died at age 2 and would have lived to age 68, producing 66 years of life saved for each child who lived. For each mother who died, we assume conservatively that she died at age 30 and would have lived to age 80, producing 50 years of life saved for each mother who lived. While time discounting at 3% per year was advocated when DALYs were first introduced, the World Health Organization discontinued this in 2010 [24]

^c^We assume conservatively that the mothers and children whose lives were saved would have lived their lives without any disabilities

#### How many disability-adjusted life years (DALYs) were averted and at what cost?

Again, as shown in Table 4, we estimate conservatively that the Project would have saved 2,146 years of life for each year of operation in the entire Project Area, and the cost per year of life saved would have been $265. Since we assume conservatively that no disabilities would be experienced by those whose lives were saved, then the number of DALYs averted is the same as the number of years of life saved: 2,146. The cost per DALY averted would be the same as the cost per year of life saved: $265.

#### Does the cost per DALY averted qualify the project as a cost-effective intervention?

The per capita gross national income (GNI) for Guatemala in 2015 was $3,700 [20]. Current recommendations are that an intervention is cost-effective if the cost per DALY averted is less than one-half of the per capita GNI for the country in which the intervention has been implemented [21, 22]. The estimated cost per DALY averted by the Project ($265) is only 7% of the per capita GNI for Guatemala. Thus, according to these estimates, the Project is a highly cost-effective intervention.

## Discussion

The implementation research reported in this series of papers provides evidence of the effectiveness of the CBIO+ Approach as implemented by Curamericas/Guatemala in the Department of Huehuetenango Project Area in (1) improving the population coverage of key evidence-based interventions designed to address epidemiological priorities, (2) reducing child and maternal mortality, (3) reducing undernutrition in children younger than 2 years of age, and (4) empowering women and communities to improve their own health. The Project was able to involve *comadronas* in the provision of maternity care at Community Birthing Centers and thus integrated them into the formal health system*.* The staff and other key stakeholders considered CBIO+ to be a feasible and effective approach. Finally, it is a cost-effective approach as judged solely by the cost of achieving its mortality impact.

There are few evaluations of population-based integrated maternal and child health program in a resource-limited setting published in the peer-reviewed literature that are as comprehensive as this one, measuring (1) changes in population coverage of multiple interventions, (2) changes in child nutritional status, (3) changes in maternal and child mortality, (4) management of obstetrical complications at the local level, (5) empowerment of women as a result of engagement with the program, (6) an assessment by staff and key stakeholders of the strengths and weaknesses of the program, and (7) an assessment of the cost-effectiveness of the program. A notable recent comprehensive evaluation of a large-scale population-based maternal and neonatal program in Uganda and Zambia that included a mortality impact and cost-effectiveness assessment has recently been published [25–28]. The implementation research reported in our series has the potential to serve as a useful guide for future implementation research aimed at reducing inequities in maternal and child health.

This evaluation is also a valuable addition to the previously published review of evidence concerning the effectiveness of community-based primary health care in improving maternal and child health [29]. That review noted the paucity of evidence regarding the effectiveness of projects and programs in populations of more than 25,000 mothers and children for more than three years, and most studies assessed only one or only a few interventions [29]. The Curamericas Maternal and Child Health Project reported in this series, in contrast, has implemented a wide range of interventions and has used multiple approaches to measuring their effectiveness over a period of four years for 48,000 mothers and under-2 children.

The effectiveness of CBIO+ could be further enhanced by (1) the expansion of Community Birthing Centers so that all women have a readily available local birthing center and also a readily available site for first-level primary health care services, (2) the resumption of the government’s *Programa de Extensión de Cobertura* (Extension of Coverage Program, or PEC) to provide basic primary health care services by ambulatory nurses who visit isolated communities monthly, and (3) the creation of a community health worker cadre that is trained and authorized to provide evidence-based interventions recommended by the World Health Organization (WHO) and UNICEF in resource-limited settings for home-based neonatal care [30], community-case management of childhood illness [31], and distribution of misoprostol to women who plan to give birth at home [32].

### The added value of CBIO

Inherent in the Census-Based, Impact-Oriented Approach are principles that are essential for reducing inequities in maternal and child health, namely (1) working in partnership with communities toward the goals of improving the health of geographically defined populations and responding to the health priorities of communities as they define them, (2) working with the community to identify all households and everyone living in each household and to maintain routine contact with everyone, (3) using locally acquired data to identify those with the greatest health needs, define program priorities, and assess progress in improving health. Part of the power of CBIO is that it is applicable to any population anywhere in the world. Even in the most advanced societies, there are epidemiological priorities – the most serious, readily preventable or treatable conditions within the population – that need to be addressed in order to improve population health. CBIO provides a long-term, holistic lens through which to envision health-related activities that can lead to meaningful improvements.

### The added value of Care Groups

Care Groups have been used effectively in many settings to improve the coverage of key maternal and child health interventions [7, 33–35], and the Project described here is yet another example. The effectiveness of the Care Group Approach in expanding the population coverage of key maternal and child health interventions has been demonstrated to surpass that of the other child survival projects supported by the United States Agency for International Development (USAID) and its Child Survival and Health Grants Program (CSHGP) that did not use the Care Group Approach [36]. The Care Group Approach provides an ideal community-based platform for health education and behavior change at the household level.

Care Groups also provide a platform onto which Positive Deviance/Hearth workshops [37] can be readily established. The PD/Hearth intervention, described in Paper 4 of this series [14], confirmed that even in the food insecure context where the Project was operating, there were available and affordable nutritious foods that could alleviate undernutrition if properly included in a child’s diet. The PD Hearth intervention contributed to the Project’s success in reducing undernutrition.

### The added value of Community Birthing Centers *(Casas Maternas Rurales)*

The Community Birthing Center Approach used by Curamericas/Guatemala is unique, innovative, and worthy of further development. One recent report describes a similar approach that was unsuccessful [38]. In that report, a birthing center was established adjacent to a hospital in the highlands of Chiapas, Mexico, not far from the Project site and in a region very similar to our Project Area – rural, mountainous, and populated by indigenous Maya people with limited access to health services and a long history of discrimination and marginalization. In stark contrast to our birthing centers, the edifice was constructed by the Mexican government without consulting with the local communities for whom they were intended and without their participation in the construction or management. Little community outreach was done by the staff of the birthing center there, and the staff members were not local or Maya. The local traditional birth attendants, known there as *parteras*, were not contacted, let alone integrated into the functioning of the birthing center. As a result almost no local women chose to deliver in the birthing center, and when interviewed for the study the women showed little understanding of its purpose and low trust in its services. This experience demonstrates the value of the CBIO+ Approach in creating Community Birthing Centers on the foundation of CBIO and Care Groups to engage communities, build trust, and establish partnerships from the outset.

Our verbal autopsies clearly indicate that one of the main reasons cited by families for not bringing women with delivery complications or bringing sick children to government health facilities is the fear of disrespectful and discriminatory treatment. A recent study in a nearby area of the Western Highlands confirmed this pervasive disrespect and abuse of Indigenous women by non-Indigenous health facility staff [39]. Our birthing centers, with their respectful and culturally-appropriate services provided in the women’s native language are clearly responding to this issue, not only through their “de-medicalization”/humanization of services, but also by providing high-quality care. These two reasons were repeatedly cited by local women as key reasons they chose to have their babies delivered in the Community Birthing Center [10]. The inclusion of *comadronas* as integral members of the staffs of the Community Birthing Centers helped to promote cultural sensitivity there and to motivate mothers to use the Birthing Center*.*

### Is the CBIO+ Approach cost-effective?

For a total cost of $14.05 per beneficiary per year, $5.80 per capita per year, and $257 per year of life saved and per DALY averted, the CBIO+ Approach is highly cost-effective purely on the basis of its reduction in maternal and child mortality without taking into account its other notable benefits for improved nutrition, women’s empowerment, and enhanced social capital. Can the government of Guatemala afford to finance such a program? At the end of the Project in 2015, Guatemala’s total health expenditure per capita for health care, including both public and private sources, was $222 [40]. The same year, the Government of Guatemala spent $84 per capita for health care nationally [41]. Thus, it appears that $5.60 per capita spent per year for high-risk, difficult-to-reach populations that are national priorities for health improvement is an investment that is affordable for the Guatemalan government [42].

The CBIO+ Approach is effective, affordable and cost-effective for Guatemala, thus meriting consideration for scale-up in other parts of Guatemala as well as for implementing and testing the CBIO+ Approach in areas of the world where resources are highly constrained, access to healthcare is difficult, health services are limited, and the burden of disease among children and mothers remains high.

### The need for approaches specifically adapted to difficult-to-access mountainous locations

The Project was implemented in a difficult-to-access mountainous location. Such locations pose serious challenges to health care delivery and are a well-documented marker of health inequity due to geographic constraints. As such, strategies for strengthening health services in these contexts merit special attention. A recent systematic literature review of effective approaches in these contexts [43] identified four broad categories: (1) using trained and supported community health workers to distribute critical medicines to the home, (2) improving the desirability of existing health services by strengthening training and supervising of health workers and the quality of care they provide, (3) generating demand for health care utilization through community engagement, and (4) improving health knowledge for timely care seeking. These are all elements that were built into the CBIO+ Approach in the Project Area.

### USAID child survival and health grants program and its contribution to the project

The methods and approaches used for this Project and the implementation research associated with it were developed in part as a result of the support provided to Curamericas Global and to the international NGO community more broadly by USAID through its Child Survival and Health Grants Program (CSHGP). Over its more than three-decade lifetime, from 1985 to 2017, the CSHGP made many important contributions to the improvement of the health of mothers and children throughout the world. First, it saved lives by supporting 465 child survival projects implemented by US-based international NGOs that expanded the population coverage of evidence-based child survival interventions implemented at the community and household levels. These projects reached 221 million children and their mothers and a population of more than 1 billion people [44].

The experience that Curamericas Global had in implementing these grants contributed to the development of the CBIO and Care Group Approaches. Curamericas Global benefitted from a number of grants from this program, beginning with its first grant to Andean Rural Health Care in 1987 and followed by other grants for Bolivia, Guatemala and Liberia. The grant that supported the Curamericas/Guatemala Maternal and Child Health Project, 2011–2015, was one of the last made by the CSHGP. Features of the grants given by the CSHGP that were consistent over the three decades of its existence were the following:

- Household knowledge, practice, and coverage surveys to measure baseline and endline population coverage of key evidence-based child survival indicators;
- The utilization of standardized indicators for measuring the population coverage of evidence-based interventions; and,
- The preparation of a detailed implementation plan that underwent peer review during the first year of the project based on the findings of the baseline household survey.

The CSHGP contracted with technical experts to provide support to the international NGOs implementing the grants and enabled program managers, who were required to have a master’s degree in an appropriate field, to meet annually to discuss their projects, learn from each other, and hear about technical advances from experts in the field. Curamericas Global and its predecessor, Andean Rural Health Care, all benefitted from these opportunities afforded by the CSHGP. All of these opportunities afforded by the CSHGP assisted in the gradual development of the CBIO+ Approach. In addition, the USAID CSHGP supported the 1993 analysis of the CBIO Approach [45] and the work of an Expert Review Panel to assess the potential of CBIO for further development and implementation [46].

The Care Group Approach was in many respects a product of the CSHGP in the sense that one international NGO (World Relief) developed the Care Group Approach as part of one of its child survival grants from the CSHGP, and Curamericas Global was the second organization to implement the Care Group Approach. The effectiveness of the Care Group Approach was quickly recognized through the results it achieved in terms of expansion of population coverage of evidence-based child survival interventions. This was possible by comparing project evaluations that measured changes from baseline to endline in population coverage in key child survival interventions, using similar methodologies (i.e., the same KPC methodology and the same indicator measurements) [36]. Also, because grantees were meeting annually to discuss their projects, enthusiasm for the Care Group Approach grew quickly. Success motivated a growing number of international NGOs to implement the Care Group Approach [7].

### CBIO+: Quo vadis?

The Expert Review Panel, convened in 1993 by the CSHGP to assess the CBIO Approach, consisted of prominent individuals from academia including from the Johns Hopkins School of Public Health, international health organizations including UNICEF, the Population Reference Bureau, and USAID; and international NGOs including Care, Save the Children, Project Hope, and Plan International. The Panel held multiple face-to-face meetings and sent two representatives to Bolivia to observe directly the implementation of the CBIO Approach there. The Panel concluded that the “[CBIO] Approach merits support by AID-Bolivia and by health development agencies in other countries as an important strategy for improving health, especially where child survival initiatives have increased community understanding of the effectiveness of modern health care” ([46]^p.9^).

At the same time, the Panel raised questions about the sustainability of the approach since it relied on auxiliary health nurses who were better paid than those in the Bolivian Ministry of Health. The Panel also noted that the communities were not as engaged as would have been desirable. Thus the two main criticisms levelled by the Panel were the need to reduce costs and engage the community further rather than providing services at the doorstep with minimal expectations on the part of the beneficiaries. Overall, then, the net effect of the Expert Review Panel’s recommendations was lukewarm. No further funding emerged to expand the implementation of CBIO in other places by other programs, as had been hoped.

The emergence of the Care Group Approach in the 2000s was in certain respects the answer to the limitations of CBIO that had been identified by the Expert Review Panel a decade earlier. Care Groups are to a notable degree a form of CBIO “lite” – that is to say, Care Groups implement a number of important CBIO principles that include establishing community partnerships, making routine systematic home visits, addressing epidemiological priorities, and monitoring mortality impact through registration of vital events. Since the implementers of the Care Group Approach are volunteers rather than paid auxiliary health nurses, as was the case with the implementation of the CBIO Approach in Bolivia, and since Care Groups require active participation of the community, it became apparent that Care Groups were a useful and, in fact, a highly valuable addition.

As an approach, Care Groups have been widely adopted, mostly by international NGOs, throughout the world [7], and the effectiveness of the approach in expanding population coverage of key child survival interventions and reducing under-5 mortality has been well-documented [35]. However, Curamericas/Guatemala is the first organization to formally link Care Groups with the CBIO Approach. The additional elements of CBIO that are not in the Care Group Approach, as it is typically implemented, include formulating a community diagnosis by working with the community to define program priorities based on the local epidemiological priorities as defined by local data and the community’s own perceived priorities, the joint development of a program plan based on the community diagnosis, and the repetition of the cycle every 4–5 years.

The lack of interest in implementing the CBIO Approach in spite of its demonstrated benefits has been a bit of a mystery, but several factors may have been at play. Among them are the following:

- CBIO is a complex intervention with many different moving parts and thus is not readily implemented without strong, highly competent, and deeply committed leadership.
- CBIO is a long-term, comprehensive intervention that requires a minimum of 5–10 years in order to demonstrate notable progress. Therefore, CBIO is not attractive to donors, who unfortunately often have a narrow, short-term focus on one or only several narrowly defined interventions.

Given this history, it seems reasonable to ask, is there really a future for CBIO+ ? We remain hopeful for several reasons. First, disparities and inequities in health status are persisting, partly because health programs have often “picked the low-hanging fruit” by focusing on narrowly defined, high-impact interventions such as vitamin A, immunizations, community case management of childhood illness, family planning, HIV programming, and so forth. After all, the seminal call for focusing on selective primary health care by Walsh and Warren in 1980 [47] referred to the selective approach in their sub-title as an interim strategy for disease control. In contrast, by engaging communities, building trust and establishing partnerships from the outset of the program, CBIO+ can provide the necessary comprehensive approach to long-term improvents in the health of populations in resource-limited settings.

Second, there is renewed interest in strengthening community health worker programs throughout the world [48] and a growing role for routine systematic home visitation as a foundational element of the work of community health workers [49]. This will make it easier to incorporate CBIO principles into these programs.

Third, evidence matters. Approaches that are demonstrated to be effective will eventually be replicated, and if their effectiveness remains, implementation will expand. This process takes time. Thus, we remain optimistic that the approach will gain steam given our findings from the Project described in this series.

### Limitations of the study

Limitations of aspects of this implementation research are reported in the previous papers of this series, including the following:

1. The Project was too brief for the CBIO+ Approach to achieve its full impact,
2. The premature and abrupt closure of the governments PEC Program diminished the Project’s impact,
3. The lack of an accurate baseline of vital events prevented the measurement of the Project’s full impact on the reduction in child and maternal mortality,
4. The lack of a true comparison area wholly outside the Project service area led to an inability to assess the Project’s impact more rigorously,
5. Some indicators, including those measuring women’s decision-making participation and community solidarity, were imperfectly defined, which limited the measurement of the Project on these outcomes.

### Evolution of Curamericas/Global and Ministry of Health activities since the completion of the Project in September 2015

Eight years have elapsed since the Project ended. In spite of concerted efforts to find additional funding to sustain Project operations in 2015, Curamericas Global and Curamericas/Guatemala had to reduce its operations and currently provides the same program support for a reduced population in the Project Area. However, with minimal additional funding from Curamericas but strong support from the Guatemala *Ministerio de Salud Pública y Asistencia Social* (MSPAS/Ministry of Health and Social Assistance) at the departmental level, Curamericas has been able to establish three new Community Birthing Centers in the Departments of San Marcos. Unfortunately, aside from this, the MSPAS activities in the Project area remain essentially unchanged since the termination of the Project, and the PEC Program still has not yet restarted.

### Implications of the results and recommendations for programs and policy.

CBIO+ merits further implementation, evaluation, and scale-up in Guatemala in partnership with the Government of Guatemala, municipal governments, and the NGO community. The ability of the CBIO+ Approach to reduce child and maternal mortality, especially from the prime causes – namely childhood pneumonia, early neonatal conditions, and postpartum hemorrhage – could be strengthened. This can be done by (1) converting Community Birthing Centers into full-service, culturally appropriate, and physically accessible rural clinics (*Centros de Atención Primaria*) that are formally recognized and supported financially by MSPAS to compensate for the lack of such services at present; (2) hiring and training community health workers to manage childhood illness, including pneumonia as recommended by WHO [31]; (3) visiting the homes of newborns frequently during the neonatal period to reinforce appropriate neonatal care behaviors and identify neonates in need of treatment and/or referral as recommended by WHO [30], and (4) enabling community health workers to distribute misoprostol to pregnant women delivering at home in order to reduce the risk of postpartum hemorrhage as recommended by WHO [32]. Of course, a stronger peripheral health system with more readily available higher-level health staff to provide preventive, promotional and curative care (including family planning services) would be helpful too in reducing mortality, but these services were beyond the scope of the Project.

The Project’s well-developed vital events registration system needs further support to capture all of the vital events in the Project population. This system has the potential of serving as a model for strengthening vital events registration throughout the country, not only for civil registration purposes but also for local program planning and monitoring.

With further refinement and ongoing efforts, the Project can provide leadership in Guatemala and beyond in creating “bottom-up” community-oriented models for improving population health. Such approaches are greatly needed not only in Guatemala but also throughout the world by serving as a complement to “top-down” programs. As demonstrated in this series, the effectiveness of CBIO+ in improving the health and well-being of mothers and children as well as the cost-effectiveness of the approach make it an important strategy for further development and broader implementation, not only in hard-to-reach populations as in Guatemala but also in other resource-constrained settings with a high burden of maternal and child mortality, including in urban settings where a growing percentage of the world’s population and poor live.

**Fig. 1:**
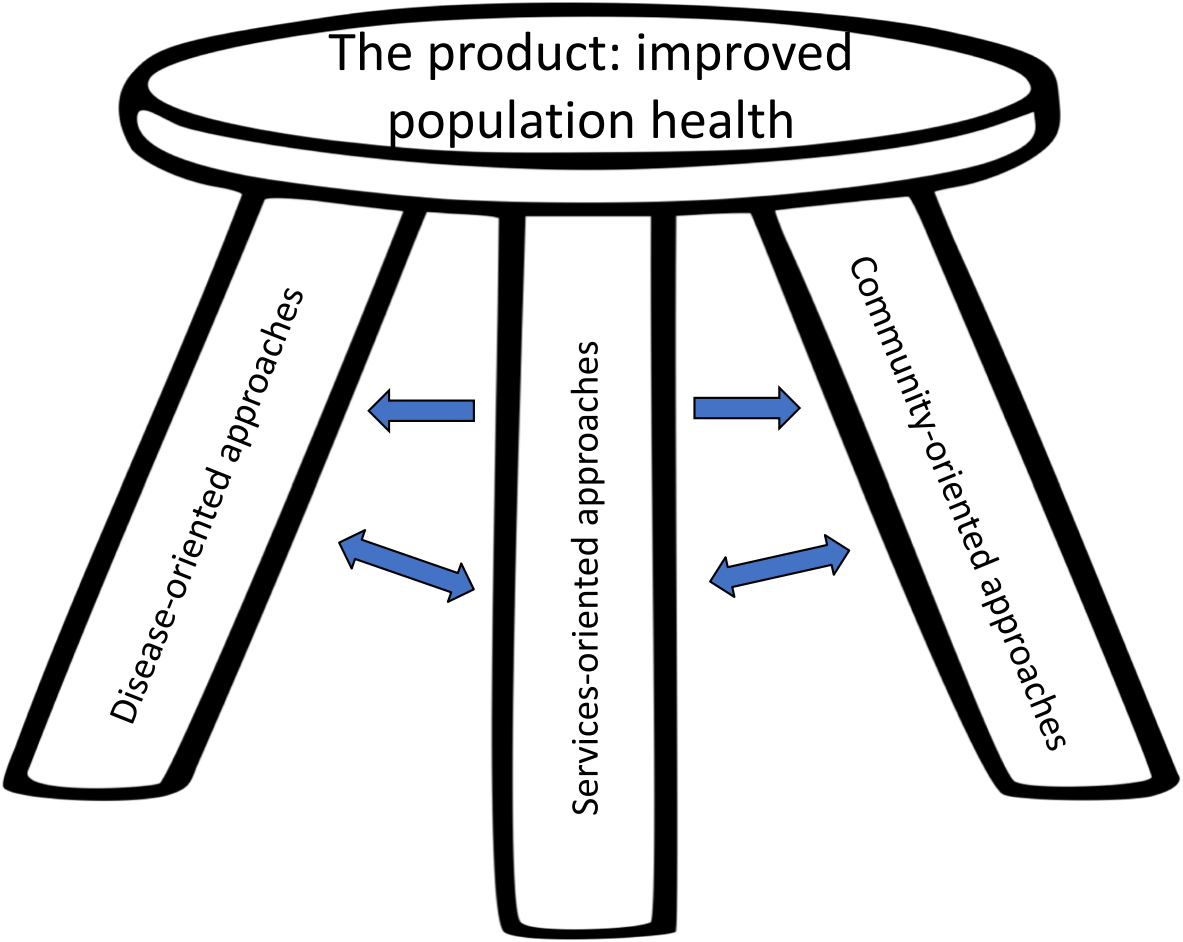
The value of a balanced approach to public health programming that includes a strong component of community engagement

Our findings serve to highlight the importance of community-oriented public health as a valuable strategy to improve the health of geographically defined populations. The goal of community-oriented public health is to work with communities to help them improve their health. Community-oriented public health complements and strengthens the effectiveness of disease-oriented public health, whose goal is to ameliorate the effects of specific diseases, and services-oriented public health, whose goal is to get basic and essential services to those who need them. When each of these components of public health programming are well-developed, the program’s foundation and, therefore, its effectiveness is enhanced, as shown in Fig. 1.

A paradigm change is needed, not only in the rural highlands of Guatemala but also in other underserved areas around the world to develop tailored approaches along the lines of what Curamericas/Guatemala has developed. The achievement of Universal Health Coverage, ending preventable child and maternal deaths, and eventually achieving Health for All will ultimately depend on working in partnership with communities to help them improve their health. CBIO+ has relevance as a feasible strategy for achieving Universal Health Coverage in high- and middle-income countries as well as in low-income countries. As Rasanathan and Diaz concluded in their 2016 commentary [50]:

The case for action on social determinants and health inequities has well and truly been made. The community of researchers on health equity now need to turn their attention to supporting implementation efforts towards achievements of the Sustainable Development Goals and substantive reductions in health inequities.

## Conclusion

The findings from this implementation research will add to the emerging, but still limited, evidence regarding the effectiveness of the CBIO+ Approach in addressing inequities in maternal and child health. This approach shows promise and, as such, deserves expanded application by Curamericas/Guatemala and other organizations in Guatemala as well as application more broadly beyond Guatemala with ongoing rigorous evaluation, further adaptation, and expansion. The methods used in this implementation research deserve broader use for strengthening primary health care programs around the world.

## Data Availability

All of the Project reports, de-identified data, as well as publications about the Expanded CBIO+ Approach cited in this article are available from the corresponding author on request.

## Abbreviations

CBIO: Census-Based, Impact-Oriented Approach
CBIO+: The combination of the CBIO Approach with Care Groups and Community Birthing Centers (*Casas Rurales Maternas*)
CSHGP: Child Survival and Health Grants Program
KPC: Knowledge, practice, and coverage (household survey)
LMICs: Low- and middle-income countries
M&E: Monitoring and evaluation
MSPAS: *Ministerio de Salud Pública y Asistencia Social* (Ministry of Health and Social Assistance)
PEC: *Programa de Extensión de Cobertura* (Extension of Coverage Program)
SIAS: *Sistema Integral De Atención En Salud* (Integrated System of Health Services)
USAID: United States Agency for International Development
